# Multiple myeloma cancer stem cells

**DOI:** 10.18632/oncotarget.8154

**Published:** 2016-03-17

**Authors:** Minjie Gao, Yuanyuan Kong, Guang Yang, Lu Gao, Jumei Shi

**Affiliations:** ^1^ Department of Hematology, Shanghai Tenth People's Hospital, Tongji University School of Medicine, Shanghai, China

**Keywords:** myeloma stem cells, surface marker, drug resistance, signaling pathways, microRNA

## Abstract

Multiple myeloma (MM) remains incurable despite much progress that has been made in the treatment of the disease. MM cancer stem cell (MMSC), a rare subpopulation of MM cells with the capacity for self-renewal and drug resistance, is considered to lead to disease relapse. Several markers such as side population (SP) and ALDH1^+^ have been used to identify MMSCs. However, ideally and more precisely, the identification of the MMSCs should rely on MMSCs phenotype. Unfortunately the MMSC phenotype has not been properly defined yet. Drug resistance is the most important property of MMSCs and contributes to disease relapse, but the mechanisms of drug resistance have not been fully understood. The major signaling pathways involved in the regulation of self-renewal and differentiation of MMSCs include Hedgehog (Hh), Wingless (Wnt), Notch and PI3K/Akt/mTOR. However, the precise role of these signaling pathways needs to be clarified. It has been reported that the microRNA profile of MMSCs is remarkably different than that of non-MMSCs. Therefore, the search for targeting MMSCs has also been focused on microRNAs. Complex and mutual interactions between the MMSC and the surrounding bone marrow (BM) microenvironment sustain self-renewal and survival of MMSC. However, the required molecules for the interaction of the MMSC and the surrounding BM microenvironment need to be further identified. In this review, we summarize the current state of knowledge of MMSCs regarding their phenotype, mechanisms of drug resistance, signaling pathways that regulate MMSCs self-renewal and differentiation, abnormal microRNAs expression, and their interactions with the BM microenvironment.

## INTRODUCTION

Multiple myeloma (MM) is a hematologic cancer characterized by proliferation of clonal plasma cells in the bone marrow (BM) resulting in the secretion of monoclonal immunoglobulins [1, 2]. MM is the second most common hematologic malignancy with an incidence ranging from 4 to 6 per 100,000/year in the USA [3–5]. Together with autologous stem cell transplantation and advances in supportive care, the introduction of novel drugs such as proteasome inhibitors and immunomodulatory drugs have significantly improved response rates and survival rates in patients with MM [6, 7]. However, MM remains incurable. Cancer stem cells (CSCs) are believed to be the major cause of tumor recurrence [8, 9]. Although the concept of CSCs has been around for decades, CSCs were only definitely described in 1997 in acute myeloid leukemia when Bonnet et al. noticed that a rare population of CD34+CD38- possess tumorigenic potential [10]. Subsequently, CSCs were identified in various solid and hematological tumors, including MM [11–15]. Although much progress in the biology and treatment of MM cancer stem cell (MMSC) has been made, there are still many questions to answer, such as what is the MMSC phenotype, what is the precise mechanism of their drug resistance, what are the precise roles and relationships of signaling pathways involved in maintaining the properties of MMSCs, what are the roles of microRNAs in MMSCs, and what are the required molecules for the interaction of the MMSC and the surrounding BM microenvironment. Further elucidation of the characteristics of MMSCs could lead to innovative strategies that could make MM a curable disease.

## MMSCS CONCEPTS

Although many investigations have tried to identify the MMSCs phenotype, there is still controversy on the MMSCs phenotype. The major viewpoint is that the clonotypic CD138^−^ cells represent MMSCs [16]. However, some investigations demonstrated that clonotypic CD138^+^ plasma cells have the properties of CSCs such as self-renewal, tumour-initiating potential and drug resistance [17]. Besides these two viewpoints, some investigators found that the interconversion between undifferentiated and differentiated clonotypic cells might be responsible for maintaining the properties of MMSCs [18]. The exact reasons for the discrepancies in reported MMSC phenotypes are unclear. It is likely that differences between clonogenic assays contribute to these disparate findings. In addition, it is possible that MM represents a number of biologically distinct diseases each containing different stem cells. Side population (SP) cells have generally been accepted to have characteristics of CSCs. They have been widely used to sort some types of CSCs of which phenotype has not been defined [19, 20]. In myeloma research, SP cells are the important source to research MMSCs.

### Clonotypic B cell

Matsui et al. [16] found that CD138^−^ cells from both human MM cell lines and primary patient samples had greater clonogenic potential both *in vitro* and in non obese diabetic/severe combined immunodeficiency (NOD/SCID) mice, compared to corresponding CD138^+^ plasma cells. Furthermore, these CD138^−^ cells were able to differentiate into CD138^+^ plasma cells and phenotypically resembled postgerminal center B cells, and their clonogenic growth could be inhibited by the anti-CD20 monoclonal antibody rituximab. These data imply that CD138^−^ B cells contained the properties of MMSCs. Matsui et al. [21] further found that CD138^−^ B cells were resistant to clinical anti-MM agents (dexamethasone, lenalidomide, bortezomib, and 4-hydroxycyclophosphamide) and possessed a high drug efflux capacity and intracellular drug detoxification activity. They also found that CD19^+^CD27^+^CD138^−^ with a memory B-cell phenotype could engraft NOD/SCID mice during both primary and secondary transplantation. Furthermore, both the side population and Aldefluor assays were able to identify CD19^+^CD27^+^CD138^−^ B cells within the peripheral blood of patients with MM. Boucher et al. [22] reported that CD19^+^CD34^+^ immature B cells and CD19^+^CD34^−^ mature cells, but not CD19^−^CD34^+^ cells isolated from the BM of patients with MM showed colony formation activity and resistance to melphalan, lenalidomide, and bortezomib, indicating undifferentiated clonotypic B cells may represent MMSCs. Kirshner et al. [23] presented a 3-D culture model in which the human BM microenvironment was reconstructed *in vitro*. In the 3-D model, drug-resistant CD20^+^ cells exhibited self-renewal potential, and gave rise to clonotypic B and plasma cell progeny in colony assays. The above studies imply that clonotypic B cells may be involved in the MM disease but offer no definitive evidence that clonotypic B cells represent the MMSCs.

### Clonotypic non B cells plasma cells

Many studies also have documented the clonogenic potential of non B cell plasma cells. Yaccoby et al. demonstrated that CD38^++^CD45^−^ MM plasma cells grew and produced MM and its manifestations in severe combined immunodeficiency (SCID) mice implanted in human fetal bone (SCID-hu mice) [24] or in rabbit femurs (SCID-rab mice) [25]. In contrast, the plasma cell-depleted BM cells did not grow or produce MM in SCID-hu mice. Similarly, plasma-cell-containing blood cells grew and developed into MM disease in SCID-hu mice, while the plasma cells depleted of blood cells weren't able to grow in SCID-hu mice, demonstrating the proliferative potential of MM plasma cells. Kim et al. [17] identified MM-initiating cells by transplanting fractionated BM cells from patients with MM into human bone-bearing immunocompromised mice. They found that CD38^high^ /CD138^+^ cells repopulated B-lineage cells in human bone grafts, and these grafts were clonally derived from patient myeloma cells. In their experiment models, only fully differentiated CD38^high^/CD138^+^ plasma cells were capable of engrafting and serially transferring the disease to secondary recipients, indicating fully differentiated CD38^high^/CD138^+^ plasma cells are enriched in myeloma-initiating cells. Hosen et al. [26] reported that CD138^−^CD19^−^CD38^++^ plasma cells were able to form MM colonies *in vitro*, while CD19^+^ B cells never formed MM colonies in 16 samples examined in their study. Furthermore, CD138^−^CD19^−^CD38^++^ plasma cells isolated from 3 out of 9 patients engrafted in the SCID-rab model produced MM, while CD19^+^ B cells did not induce MM growth. CD138^+^ plasma cells isolated from 4 out of 9 patients developed MM, although more slowly than CD138^−^ cells. Finally, CD19^+^ B cells from 13 patients with MM transplanted into NOD/SCID IL2Rγc^−/−^ mice did not propagate MM. Their data showed that in some patients with MM plasma cells but not B cells were enriched with CD138^−^ clonogenic cells, and that MM plasma cells could develop MM *in vivo* in the absence of CD19^+^ B cells. Paino et al. [27] examined in several MM cell lines the presence and functionality of CD20^+^ putative MMSCs. Only a very rare population of CD20^dim+^ cells (0.3%) in the RPMI-8226 cell line was detected. Furthermore, CD20^dim+^ RPMI-8226 cells were not essential for CB17-SCID mice engraftment and had lower self-renewal capacity than the CD20^−^ RPMI-8226 cells. Their data showed that CD20 may not be a marker of MMSCs. Trepel et al. [28] established a novel approach that directly tracked clonotypic B cells in 15 patients with MM. They found clonotypic B cells in only one out of 15 patients with MM, indicating clonotypic B cells represent a very small population in MM. Chiron et al. [29] showed that the peripheral CD138^+^CD20^−^ population contains MMSC activity in patients with plasma cell leukemia, which is an aggressive presentation of MM with high-level proliferation. They further found that this population supported the establishment of human MM cell lines.

### Phenotypic and functional plasticity between undifferentiated and differentiated clonotypic cells

The unidirectional hierarchical model from undifferentiated cells to differentiated cells ignores now available data that shows differentiated MM plasma cells possess a clonogenic capacity. Jakubikova et al. [30] found that SP cells express CD138 antigen in MM cell lines, indicating CD138^+^ differentiated cells have clonogenic capacity. There is growing evidence of interconversion between undifferentiated and differentiated clonotypic cells and these might be present and responsible for phenotypic diversities and maintaining of MMSCs features [18, 31, 32]. Chaidos et al. [18] showed that CD19^−^CD138^+^ plasma cell (PC) and CD19^−^CD138^−^ cell (termed Pre-PC) represent reversible, bidirectional phenotypic and functional states and share MMSC activity. In their experiment, 9 of 12 MM patient-derived highly purified CD138^high^ PCs displayed bone marrow engraftment, which is able to engraft in secondary transplants, indicating CD138^+^ PCs possess MMSCs activity. Additionally, both Pre-PCs and CD138^+/low^ PCs were identified in BM of mice receiving highly purified CD138^ high^ PCs, strongly supporting a PC to Pre-PC transition. When they assessed the drug resistance of PCs and Pre-PCs, they found Pre-PCs are much more drug-resistant than PCs although both PCs and Pre-PCs excluded vital dye in an equally efficient manner. These findings were very attractive and imply phenotypic and functional plasticity between undifferentiated and differentiated clonotypic cells. The plasticity could better explain why differentiated MM plasma cells possess a clonogenic capacity and also reconcile inconsistencies among the MM stem cell phenotype.

### SP cells

SP cells were originally identified in murine BM and referred to cells with the capacity to efflux the fluorescent dye Hoechst 33342 [33, 34]. SP cells have been identified in a variety of tissues and cancer cell lines. These cells share characteristics of CSCs, specifically, they possess tumorigenic potential, express stem-like genes, and resist to chemotherapeutic drugs [20]. This is why SP cells are believed to play a critical role in tumour maintenance and reoccurrence. Loh et al. [35] found that SP cells were present in MM cell lines as well as primary MM cells. Matsui et al. [21] showed that in human MM cell lines, SP cells were almost exclusively CD138^−^. However, Jakubikova et al. [30] reported the conflicting results that SP cells were mainly present in CD138^low+^ and CD138^+^ populations in MM cell lines, suggesting that SP cells express CD138 antigen. Moreover, they found no correlation between expression of CD19, CD20, or CD27 and the proportion of SP cells. These data were supported by the findings of Chaidos et al. [18] that showed MMSC activity is a property shared by both CD19^−^CD138^−^ cells and CD19^−^CD138^+^ plasma cells but not CD19^+^CD138^−^ clonotypic B cells. SP cells exhibit substantial heterogeneity in a panel of MM cell lines and primary MM cells; Moreover, SP cells revealed a higher tumorigenicity and proliferation index than main population (MP) cells [30]. Although results regarding SP cells seems to be convincing, the approach for selection of SP has been questioned. The dye Hoechst 33342 has lethal effect on living cells, which is why SP cells might represent only a population that survived the toxic effect of Hoechst 33342 [31]. Montanaro et al. [36] demonstrated that yield, viability, and homogeneity of SP cells were affected by the isolation parameters, such as staining time, dye concentration, cellular concentration and stringency in the selection of SP cells. In addition, they also reported that the use of verapamil as an inhibitor of efflux does not seem to be specific enough, since a small proportion of verapamil-sensitive cells are present in the SP gate.

## DRUG RESISTANCE

Conventional cytotoxic chemotherapeutic agents are capable of producing initial responses. The introduction of novel agents such as thalidomide, lenalidomide, bortezomib and carfilzomib have significantly improved clinical responses and overall survival in patients with MM [37–40]. Furthermore, synergistic effects have been clarified when these agents are used in combination treatment. Even with more available therapeutic options, a vast majority of patients with MM eventually relapse. The treatment failure demonstrates current agents are ineffective in eradicating the most drug-resistant MM cells [41]. The high drug efflux capacity of MMSCs is likely to be the major cause of drug resistance in myeloma [20, 21]. Circulating clonotypic B cells have been shown to persist and increase after chemotherapy [42–44]. In order to overcome drug resistance of MMSCs and develop innovative strategies, mechanisms of drug resistances strongly need to be clarified.

### ATP-binding cassette (ABC) transporters

ATP-binding cassette (ABC) transporters represent one family of transmembrane proteins. These transporter proteins use the energy from ATP hydrolysis to efflux cytotoxic compounds across the membrane, which is believed to be one of the major causes of multidrug resistance in cancer therapy [45]. In MM, SP cells show stronger activity of the ABC transporter ABCG2 when compared to MP cells. Moreover, inhibition of ABCG2 could decrease SP proportion. Other ABC transporters are also involved in the SP cells, an example of this is the high levels of ABCC1 were that were detected in the SP cells of the KMS-11 line. Similarly, high levels of ABCB1 were detected in SP cells of doxorubicin-resistant RPMI-Dox40 cells [30]. ABCC3 was increased in SP cells compared with MP cells in human primary MM samples, and mediated drug resistance in MM cells [46]. The expression of ABCB1 was upregulated in MM cells overexpressing bruton tyrosine kinase (BTK) and endowed these cells with drug resistance [47]. Hawley et al. [48] found that upregulation of ABCB1 expression was associated with high transporter activity. Hirschmann-Jax et al. demonstrated that expression of ABCB3 was associated with drug resistance of stem cells [49].

### ALDH

ALDH (aldehyde dehydrogenase) catalyzes the chemical transformation from acetaldehyde to acetic acid. Upregulation of ALDH expression has been detected in normal adult stem cells. ALDH is commonly deregulated in many tumors. Increased expression of ALDH in cancer is associated with increased stemness and poor clinical outcome [21, 50–54]. Matsui et al. [21] found that in RPMI 8226 and NCI-H929 cell lines, CD138^−^ cells exhibited significantly higher levels of ALDH activity than CD138^+^ plasma cells. Furthermore, they showed that majority of the ALDH^+^ B cells isolated from the peripheral blood of patients with MM express clonotypic surface Ig light chain and CD27, which were similar to the SP B cells. Our previous study demonstrated that ALDH1^+^ MM cells had much stronger capacities of proliferation and tumorigenicity, compared to ALDH1^−^ MM cells. Moreover, we found that ALDH1^+^ MM cells highly expressed chromosomal instability genes associated with drug resistance [55]. More recently, we demonstrate that member A1 of the ALDH1 family of proteins, ALDH1A1, was up-regulated in the course of myeloma therapy and progression, and that enforced expression of ALDH1A1 led to both increased tumorigenicity and resistance to two widely used myeloma drugs (doxorubicin, bortezomib) *in vitro* and *in vivo* [56]. When CSCs were treated with certain chemotherapy agents, they produced toxic aldehyde intermediates. ALDH could detoxify these toxic aldehyde intermediates. This is one of the major reasons why chemotherapy agents are not effective against CSCs expressing ALDH [57]. The number of CSCs expressing ALDH increased after chemotherapy [58]. Raha et al. [59] evaluated the role of ALDH in the maintenance of CSCs. They found that ALDH protected the CSCs from the toxic effects of elevated levels of reactive oxygen species (ROS), and that inhibition of ALDH activity decreased the proportion of CSCs and delayed treatment relapse *in vitro* and *in vivo* through accumulation of ROS to toxic levels, consequent DNA damage, and cell apoptosis.

### Increased expression of anti-apoptotic genes

Chemotherapeutic agents function by inducing apoptosis. The balance of pro- and anti-apoptotic proteins affected the apoptotic response of cells to these chemotherapeutic agents. Increased expression of anti-apoptotic proteins and decreased expression of pro-apoptotic proteins confer cells drug resistance. Our previous study demonstrated that higher expression of retinoic acid receptor alpha 2 (RARα2) was observed in the MMSC compartment and this resulted in increased drug resistance. Further research showed that the expression of anti-apoptotic Bcl-2 family members increased in MMSCs expressing RARα2 and endowed these cells with increased drug resistance [46]. More recently, we reported that expression of BTK was increased in MMSCs, and overexpression of BTK induced drug resistance, which was partially mediated by upregulation of anti-apoptotic gene BCL-2 [47].

### Cellular quiescence

Cellular quiescence is a property of hematopoietic stem cells and is thought to play a crucial role in protecting stem cells [60]. Similar to normal stem cells, CSCs have a slow cycling rate and are relatively quiescent. This property protects CSCs against chemotherapeutic agents that are effective in targeting all dividing cells [58]. This property has been conjectured to be a major mechanism of drug resistance. Matsui et al. [21] evaluated whether MM precursors are relatively quiescent. They isolated CD138^−^ MM cells and CD138^+^ MM cells from the RPMI 8226 and NCI-H929 cell lines and used propidium iodide to detect their cell cycle status. They found that the proportion of the CD138^−^ MM cells in G0-G1 is much higher than that of CD138^+^ MM cells, indicating that CD138^−^ MM cells are relatively quiescent. These results are consistent with Fuhler et al.'study. In their study, CD138^−^ MM cells have a reduced activity of kinases involved in cell cycle progression and slower G1/S phase transition compared to CD138^+^ MM cells [61]. Chaidos et al. [18] found that CD19^−^CD138^+^ PCs and CD19^−^CD138^−^ Pre-PCs fractions harbor MMSC activity and display different resistance to treatment (Pre-PCs being more drug-resistant than PCs). Both PCs and Pre-PCs lacked surface expression of the drug efflux proteins ABCB1 and ABCG2, demonstrating that ABC transporters might be not responsible for their different resistance to treatment. Further research showed that Pre-PCs were more quiescent than PCs, revealed by the lower proportion of Pre-PCs in S phase of cell cycle, suggesting that cellular quiescence may be involved in drug resistance. The above findings imply that MMSCs resistant to chemotherapeutics may be mediated by cellular quiescence.

## SIGNALING PATHWAYS

Wingless (Wnt) pathway is a classic “stemness” pathway, which plays a crucial role in normal adult stem cells as well as CSCs of many cancer types [62]. Hedgehog (Hh) pathway is typically active in hematopoietic stem cells and CSCs [63]. The Notch pathway promotes CSCs [64, 65]. These pathways are also highly active in MMSCs and may be responsible for the maintenance and sustainability of MM. Additionally, PI3K/Akt/mTOR pathway is critical for survival and has also been shown to be aberrantly active in CSCs [66]. Increased evidence demonstrates that targeting these pathways individually will not be sufficient to kill MMSCs; instead, rational combinations of agents targeting these pathways in concert could be of particular importance.

### Wnt

The Wnt signaling pathway plays a significant role in the regulation of cell proliferation, cell development and the differentiation of normal stem cells. Constitutive activation of Wnt signaling pathway is found in a variety of human cancers [67, 68]. MM cells have been reported to depend on an active Wnt signaling. The dysregulation of Wnt signaling pathways resulted in promoting MM cell proliferation, migration, invasion, drug resistance and formation of MMSCs [46]. Wnt signaling sustains stemness mainly by stabilizing β-Catenin and promoting its translocation to the nucleus where it functions as a transcription factor [69, 70]. Sukhdeo et al. [71] demonstrated that Wnt signaling pathway genes were up-regulated in MM cells. These included upstream pathway genes such as frizzled and Wnt ligands, as well as β-Catenin, TCF4, and BCL9, and a number of downstream pathway members. In their study, PKF115-584, which was a Wnt pathway inhibitor and could down-regulate Wnt target genes and block β-Catenin/TCF transcriptional activity, significantly reduced cell proliferation and inhibited tumor growth. Zhao et al. [72] reported that the microRNA miR-30-5p could reduce tumor burden and metastatic potential in murine xenograft models of MM through targeting Wnt/β-Catenin/BCL pathway. These data demonstrated that targeting Wnt signaling pathway represents a therapeutic approach in the treatment of MM. Our previous studies [46, 47] found that β-Catenin, TCF4 and LEF1 expression increased in SP cells compared to MP cells. Wnt inhibition overcame drug resistance. Moreover, Wnt inhibition reduced the myeloma tumor burden and increased survival in the 5TGM1 mouse model.

### Hedgehog

Hh signaling molecules include Hh ligands (Sonic Hh, Indian Hh and Desert Hh), the transmembrane receptor patched (PTCH), the signal transducer smoothened (SMO) and transcription factors (GLI1, GLI2, GLI3). In the absence of Hh ligand, PTCH inhibits SMO and renders the pathway inactive. When ligands bind PTCH, SMO is de-repressed and then leads to activation of the three GLI proteins that act as transcriptional regulators that regulate target gene expression. GLI1 acts as positive effector of Hh signaling and induces the transcription of Hh target genes. GLI3 acts as negative effector of Hh signaling by repressing the transcription of Hh target genes, while GLI2 can act as either positive or negative effector, depending on both post-transcriptional and post-translational modifications [73, 74]. The Hh signaling pathway is involved in the regulation of cell differentiation, proliferation, stem cell maintenance, and carcinogenesis [73, 75, 76]. Aberrant Hh signaling activation may be implicated in many types of cancer, including skin, leukemia, lung, brain, and gastrointestinal cancers [73]. Peacock et al. [77] investigated the role and mechanisms of Hh pathway activation in MM. They found that Hh pathway activity is mainly concentrated within the CD138^−^CD19^+^ MMSCs compartment but not in the CD138^+^CD19^−^ MM plasma cells. Stroma-derived Hh ligands promote proliferation of MMSCs without differentiation, while inhibition of Hh pathway markedly inhibits clonal proliferation accompanied by terminal differentiation of purified MMSCs. These data revealed that Hh pathway activation is heterogeneous between MMSCs and differentiated MM cells, and that Hh pathway activation plays a crucial role in maintaining MMSCs in an undifferentiated, clonally expansion state. More recently, Liu et al. [78] reported that CD138^+^ MM cells are a major source of Hh ligand SHH and autocrine SHH enhanced MM cells proliferation and drug resistance, indicating that the myeloma autocrine Hh signaling pathway was a promising therapeutic target in MM.

### Notch

The Notch signaling pathway is activated in MM cells, which leads to increased proliferation, resistance to apoptosis and osteoclastogenesis [79–82]. Jundt et al. [80] evaluated the pathogenetic role of Notch signaling pathway in MM. They found that Notch receptors and ligand Jagged1 were highly expressed in cultured and primary MM cells. Furthermore, Jadded1-induced Notch signaling strongly promoted MM cell growth. Nefedova et al. [83] reported that targeting Notch signaling *via* its specific inhibitorγ-secretase inhibitor (GSI) induced apoptosis of MM cells and inhibited tumor growth in xenograft and SCID-hu models of MM. Additionally, inhibition of Notch signaling enhances MM cell sensitivity to chemotherapeutic drugs such as doxorubicin and melphalan. Mirandola et al. [84] demonstrated that Notch signaling pathway plays a significant role in MM cell lines through activating CXCR4/SDF-1 axis, and that forced CXCR4 activation partially protects MM cells from the outcomes of Notch inhibition. In addition, Notch signaling blockage markedly reduces bone marrow (BM) infiltration by human MM cells in mouse xenografts. Their data showed that a Notch-targeted approach is effective in preventing MM cell migration, proliferation and survival. More recently, Colombo et al. [85] presented evidence that MM induced osteoclastogenesis by activating Notch signaling on tumor cells and osteoclasts through Jagged ligands expression. Silencing tumor-derived Jagged1 and 2 ligands could block MM-induced osteoclastogenesis. These results suggest that Notch signaling plays a key role in osteoclastogenesis and is a promising therapeutic target in MM to prevent bone disease. Great expression of Notch signaling has been detected in BM clonotypic B cells from patients with MM [86]. However, the precise roles of Notch signaling pathways remain to be further investigated in MMSCs.

### PI3K/Akt/mTOR

The PI3K/ Akt/mTOR signaling pathway is aberrantly activated in many cancer types. A preferential inhibitory effect on CSCs has been shown for some mTOR inhibitors [87, 88]. PI3K/mTOR inhibitor VS-5584 preferentially reduced the proportion of CSCs in multiple mouse xenograft models of human cancer. Mechanistic research showed that coordinate RNAi-mediated silencing of PI3Kα, PI3Kβ, and mTOR replicated the effect of VS-5584, indicating that preferential targeting of CSCs required inhibition of multiple components of the PI3K/mTOR pathway [89]. PI3K/Akt/mTOR pathway is activated and play a critical role in MMSCs. Du et al. [90] found that PI3K/Akt/mTOR signal transduction proteins CAB39, TSC1, P - S6 and P - P70S6K expression levels were significantly higher in SP cells of MM than that in MP cells of MM. Moreover, the mTOR specific inhibitor rapamycin can dramatically decrease the proportion of SP cells of MM.

## MICRORNAS IN MMSCS

MicroRNAs are small non-coding regulatory RNA molecules which inhibit the expression of their target genes at the translational level. The microRNA profile between CSCs and non-CSCs is remarkably different. Moreover, multiple microRNAs have been reported to regulate properties of CSCs [91]. Du et al. [90] isolated SP cells from MM cell lines and primary MM cells and investigated the microRNA profile between SP cells and MP cells, finding a total of 43 differentially expressed microRNAs (10 over-expression and 33 low-expression microRNAs) in SP cells. Among these differentially expressed microRNAs, five were associated with MMSCs. In addition, high expression of microRNA451 in SP cells activated PI3K/mTOR signaling pathways. Inhibiting the expression of microRNA451 induced apoptosis. Zhao et al. [72] demonstrated that downregulation of microRNA-30-5p could activate the oncogenic Wnt/β-Catenin pathway which was one of most frequently active pathways in MMSCs, implying that microRNA-30-5p may regulate formation of MMSCs. The differential microRNA profile in MMSCs made auxiliary biomarkers for the identification of MMSCs possible and their role in regulating the properties of MMSCs made them potential targets for therapy.

## INTERACTION OF MMSC WITH BM MICROENVIRONMENT

In MM, the microenvironment is comprised of extracellular matrix components including collagens, laminin and fibronectin and cellular parts including BM stromal cells (BMSCs), osteoblasts, osteoclasts and others [86]. BMSCs secrete factors including interleukin 6 (IL-6), insulin-like growth factor 1 (IGF-1), RANK ligand (RANKL), tumor necrosis factor alpha (TNF-α), vascular endothelial growth factor (VEGF), and stromal cell-derived factor 1 alpha (SDF1) [84, 92, 93]. These factors maintain normal cell function and stimulate MM cell survival. The BM microenvironment is crucial for self-renewal and survival of HSC. Interestingly, many of the signaling pathways involved in sustaining HSC regulate MM disease progression, indicating these signaling pathways could potentially be hijacked by the MMSCs for their self-renewal and survival [92, 94, 95]. BM hypoxia is required for normal hematopoiesis. Similarly, BM hypoxia is advantageous for the tumor-initiating CD45+ MM cells in 5T2MM mouse MM models [96]. The MMSC and the surrounding BM microenvironment interact in a way that sustain long-term survival of the MMSC. The identification of the required molecules for the interaction of the MMSC and the surrounding BM microenvironment will enable us to perturb their interactions and represent a significant effort to eradicate MMSC from the BM environment.

## CONCLUSIONS

**Figure 1:**
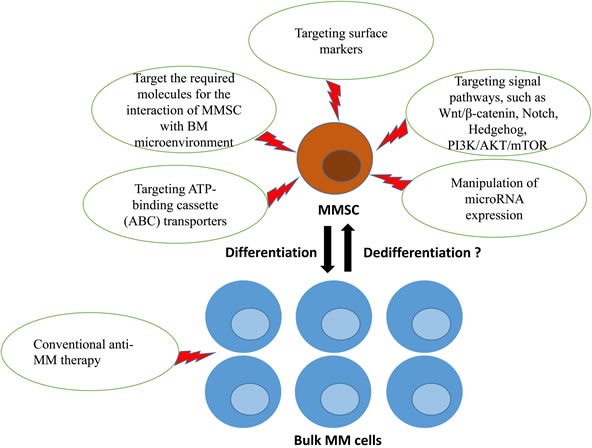
MMSCs-targeting therapy in combination with non-MMSCs therapy may offer a promising strategy for management of myeloma The promising approaches to eradicate MMSCs included: targeting surface markers, the required molecules for the interaction of MMSC with BM microenvironment, signaling pathways that regulate MMSCs self-renewal and differentiation, ATP binding cassette (ABC) transporters involved in drug resistance, manipulation of microRNA expression. Bulk MM cells might replenish the MMSC pool after this pool has been eliminated if phenotypic and functional plasticity between MMSCs and bulk MM cells exists. Consequently, curing myeloma will require a combined therapy that target both MMSCs and bulk MM cells. MM, multiple myeloma; MMSC, multiple myeloma cancer stem cell; BM, bone marrow.

In our opinion, MMSCs represent a significant effort to effective cancer treatment. However, there is still a pressing need to identify surface markers and understand molecular feature associated with MMSCs. Global gene expression profiling (GEP) is a powerful tool for identifying new targets in cancers [97, 98]. Kiel et al. used GEP to find the surface marker SLAM of leukemia stem cell [99]. Similarly, our group used GEP to find promising genes that were significantly differentially expressed in CD138^−^ cells compared to CD138^+^ cells from MM cell lines [46, 47]. In the future, the discovery of MMSC surface markers will help with the design of MMSCs-targeting drug. In addition, further studies are needed to explore the required molecules for the interaction of the MMSC and the surrounding BM microenvironment. MMSC has many similar signaling and receptors with HSC. Therefore, while understanding the molecules responsible for self-renewal and survival of the HSC in the BM microenvironment, this can be applied to the MMSC to potentially eradicate MMSC from the BM microenvironment. We speculate that MMSCs-targeting therapy in combination with non- MMSCs therapy, such as conventional anti-MM drugs, may offer a promising strategy for management and eradication of myeloma (Figure 1).
